# Reviewer acknowledgement 2014

**DOI:** 10.1186/s13071-015-0810-9

**Published:** 2015-04-11

**Authors:** Chris Arme

**Affiliations:** Editor-in-Chief, Parasites & Vectors, 236 Grays Inn Road, London, WC1X 8HB UK

## Abstract

*Parasites & Vectors* would like to thank all reviewers who have contributed to the journal in 2014.

**Rao Abbas**

Pakistan

**Ibrahim Abbasi**

Israel

**Mohd-Tajuddin Abdullah**

Malaysia

**Rabindra Abeyasinghe**

Philippines

**David Abraham**

United States

**Getu Abraham**

Germany

**Haileeyesus Adamu**

Ethiopia

**David Addiss**

United States

**Monsuru Adebayo Adeleke**

Nigeria

**Coenraad Adema**

United States

**Peter Adler**

United States

**Maria Afonso**

Portugal

**Yaw Afrane**

Kenya

**Philip Agnew**

France

**Daniel Aguiar**

Brazil

**Mariana Ahamad**

Malaysia

**Touhid Uddin Ahmed**

Bangladesh

**Rock Aïkpon**

Benin

**Nazaire Aizoun**

Benin

**Daniel Ajzenberg**

France

**Kumagai Akira**

Japan

**Munir Aktas**

Turkey

**Mohammad Shafiul Alam**

Bangladesh

**Samer Alasaad**

Spain

**Daniel Albeny Simoes**

Brazil

**Marco Albonico**

Italy

**Simon Alderton**

United Kingdom

**Abebe Alemu**

Ethiopia

**Neal Alexander**

United Kingdom

**Vahab Ali**

India

**Brian Allan**

United States

**Paulo Almeida**

Portugal

**Hesham Al-Mekhlafi**

Malaysia

**Cristina Alonso-Vega**

Bolivia

**Haoues Alout**

United States

**Salman Al-Shami**

Saudi Arabia

**Bulent Alten**

Turkey

**Cosme Alvarado-Esquivel**

Mexico

**Alfred Amambua-Ngwa**

Gambia

**Ana Amaro**

Portugal

**Ivana Amelotti**

Argentina

**Antero Andrade**

Brazil

**Andrey Andrade**

Brazil

**Helida Andrade**

Brazil

**Ana Andrade**

Brazil

**José Dilermando Andrade Filho**

Brazil

**Bennet Angel**

India

**Yadouleton Anges**

Benin

**Abebe Animut**

Ethiopia

**Brendan Ansell**

Australia

**Christophe Antonio-Nkondjio**

Cameroon

**Karim Aoun**

Tunisia

**Michelle Aparecida Ribeiro De Freitas**

Brazil

**Silvana Araújo**

Brazil

**Larry Arlian**

United States

**Frederick Arnaud**

France

**Carla Arruda**

Brazil

**Alex Asidi**

United Kingdom

**Hasan Tarik Atmaca**

Turkey

**Tatjana Avsic-Zupanc**

Slovenia

**Samson Awolola**

Nigeria

**Anna Bajer**

Poland

**Nandhakumar Balakrishnan**

United States

**Meshesha Balkew**

Ethiopia

**Claudio Bandi**

Italy

**Fu Baoquan**

China

**Ricardo Barata**

Brazil

**Bridget Barber**

Australia

**Helene Barbosa**

Brazil

**João Batista Barbosa**

Brazil

**Alan Barbour**

United States

**Beatrice Barda**

Italy

**Irka Bargielowski**

United States

**Maria Dolores Bargues**

Spain

**Walter Basso**

Switzerland

**Paul Bates**

United Kingdom

**Cici Bauer**

United States

**Melissa Beall**

United States

**Relja Beck**

Croatia

**Norbert Becker**

Germany

**Robert Beckstead**

United States

**Nigel Beebe**

Australia

**Jerzy Behnke**

United Kingdom

**John Beier**

United States

**Jean-Nicolas Beisel**

France

**Thiago Belinato**

Brazil

**Romeo Bellini**

Italy

**Paola Beraldo**

Italy

**Robert Bergquist**

Sweden

**Ulrika Bergvall**

Sweden

**Marco Bernasconi**

Switzerland

**Eduardo Berriatua**

Spain

**Nora Besansky**

United States

**Martha Betson**

United Kingdom

**Frederic Beugnet**

France

**Ian Beveridge**

Australia

**Sudipta Bhowmik**

India

**Sibhatu Biadgilign**

Ethiopia

**Jude Bigoga**

Cameroon

**Peter Billingsley**

United States

**Zeno Bisoffi**

Italy

**Andrew Blagborough**

United Kingdom

**David Blair**

Australia

**Bradley Blitvich**

United States

**Boakye Boatin**

Ghana

**Moses Bockarie**

United Kingdom

**Rene Bodker**

Denmark

**Walter Boeger**

Brazil

**Pieter Boets**

Belgium

**Christian Bogdan**

Germany

**Ken Boorom**

United States

**Mark Booth**

United Kingdom

**Ligia Borges**

Brazil

**Christian Bottomley**

United Kingdom

**Dmitri Boudko**

United States

**Emilie Bouhsira**

France

**Michel Boussinesq**

France

**Jérémy Bouyer**

Senegal

**Maha Bouzid**

United Kingdom

**Dwight Bowman**

United States

**Jan Brabec**

Czech Republic

**Janette Bradley**

United Kingdom

**Marieta Braks**

Netherlands

**Daniel Bray**

United Kingdom

**Reginaldo Brazil**

Brazil

**Emanuele Brianti**

Italy

**Olivier Johan Tavai Briët**

Switzerland

**Jory Brinkerhoff**

United States

**Claudia Brodskyn**

Brazil

**William Brogdon**

United States

**Basil Brooke**

South Africa

**Heidi Brown**

United States

**Fabrizio Bruschi**

Italy

**Alicja Buczek**

Poland

**Tullu Bukhari**

Netherlands

**Stewart Burgess**

United Kingdom

**Joan Burke**

United States

**Caroline Burri**

Switzerland

**Felicity Burt**

South Africa

**Catherine Butler**

Grenada

**Tariq Butt**

United Kingdom

**Margarita Cabrera**

Mexico

**Monica Caffara**

Italy

**Rafael Calero-Bernal**

United States

**Luis Marcelo Aranha Camargo**

Brazil

**Corey L Campbell**

United States

**Peter Campbell**

Australia

**Cinzia Cantacessi**

United Kingdom

**German Canton**

Argentina

**Gioia Capelli**

Italy

**Beniamino Caputo**

Italy

**Helene Carabin**

United States

**Luis Cardoso**

Portugal

**Yannick Caron**

Belgium

**Simon Carpenter**

United Kingdom

**Lauren Carrington**

Vietnam

**Socrates Cavalcanti**

Brazil

**Silvia Cazorla**

Argentina

**Giuliano Cecchi**

Ethiopia

**Dave D Chadee**

Trinidad And Tobago

**Elizabeth Chadwick**

United Kingdom

**Frédérique Chammartin**

Switzerland

**Kwang Poo Chang**

United States

**Theeraphap Chareonviriyaphap**

Thailand

**Johannes Charlier**

Belgium

**Remi Charrel**

France

**Luis Fernando Chaves**

Japan

**Jia-Xu Chen**

China

**Qijun Chen**

China

**Wei-June Chen**

Taiwan

**Xiaoguang Chen**

China

**Sen-Sung Cheng**

Taiwan

**Pedro Chieffi**

Brazil

**Jan Chirico**

Sweden

**Daniel Chisenhall**

United States

**Kwang Shik Choi**

South Africa

**Bruno Chomel**

United States

**Anindo Choudhury**

United States

**Valerie Choumet**

France

**Philippe Christe**

Switzerland

**Andreas Chryssafidis**

Ireland

**Ting-Wu Chuang**

Taiwan

**Peter-Henning Clausen**

Germany

**Dagmar Clough**

Germany

**Gerald Coles**

United Kingdom

**Doug Colwell**

Canada

**Hua Cong**

China

**Jan Conn**

United States

**Franz Josef Conraths**

Germany

**Anabela Cordeiro-Da-Silva**

Portugal

**Sylvie Cornelie**

France

**Stephane Cornet**

France

**Jenny Cory**

Canada

**Jane Costa**

Brazil

**Carlo Costantini**

France

**Magda Costa-Ribeiro**

Brazil

**Janet Cox-Singh**

United Kingdom

**Vasile Cozma**

Romania

**Clemente Cpasso**

Italy

**Giuseppe Cringoli**

Italy

**Zilma Cruz**

Brazil

**Feng Cui**

China

**Jing Cui**

China

**Narcisa Cunha-E-Silva**

Brazil

**Elisa Cupolillo**

Brazil

**Ana Custódio**

Portugal

**Krystyna Cwiklinski**

United Kingdom

**Hugo Leonardo Da Cunha Amaral**

Brazil

**João Aristeu Da Rosa**

Brazil

**Roch Kounbobr Dabiré**

Burkina Faso

**Jianfeng Dai**

China

**Stefano D’amelio**

Italy

**Marilvia Dansa-Petretski**

Brazil

**Filipe Dantas-Torres**

Brazil

**Jonathan Darbro**

Australia

**Michael Dark**

United States

**Murari Das**

Nepal

**Tim Day**

United States

**Jose De La Fuente**

Spain

**Claudio De Liberato**

Italy

**Wanderley De Souza**

Brazil

**Theo De Waal**

Ireland

**Alessandra Della Torre**

Italy

**Berna Demirci**

Turkey

**David Denlinger**

United States

**Marketa Derdakova**

Slovakia

**Kebede Deribe**

Ethiopia

**Wannes Dermauw**

Belgium

**Yahya Athumani Derua**

Tanzania

**Zelalem Desalegn**

Ethiopia

**Yves Desdevises**

France

**Gregor Devine**

Australia

**Sunil Dhiman**

India

**Ibrahima Dia**

Senegal

**Fernando Dias**

Brazil

**Muriel Dietrich**

Reunion

**Rod Dillon**

United Kingdom

**Pedro Paulo Diniz**

United States

**Lileia Diotaiuti**

Brazil

**Colette Dissous**

France

**Rajnikant Dixit**

India

**Brent Dixon**

Canada

**Gerhard Dobler**

Germany

**Andrew Dobson**

United Kingdom

**Michael J Doenhoff**

United Kingdom

**Stephen Doggett**

Australia

**Funda Dogruman-Al**

Turkey

**Claudia Dolinski**

Brazil

**Cristian Domsa**

Romania

**Martin Donnelly**

United Kingdom

**Gilles Dreyfuss**

France

**Arathy Ds Nair**

United States

**Joseph D’silva**

United States

**Aifang Du**

China

**Jitender Dubey**

United States

**Luc Duchateau**

Belgium

**Georg Duscher**

Austria

**Prafulla Dutta**

India

**Jan Dvorak**

United Kingdom

**Andrea Egizi**

United States

**Rebecca Eisen**

United States

**Ali Elbakri**

United Arab Emirates

**Dia-Eldin Elnaiem**

United States

**Aidan Emery**

United Kingdom

**Luchuo Engelbert Bain**

Cameroon

**Marc Engelsma**

Netherlands

**Olivier Engler**

Switzerland

**Marina Eremeeva**

United States

**Hatice Ertabaklar**

Turkey

**Nuria Escudero**

Spain

**Yuki Eshita**

Japan

**Bertha Espinoza**

Mexico

**Johan Esterhuizen**

Uganda

**Agustin Estrada-Peña**

Spain

**Walter Fabricio Silva Martins**

United Kingdom

**Chia-Kwung Fan**

Taiwan

**Robert Farkas**

Hungary

**Ronald Fayer**

United States

**Heather Ferguson**

United Kingdom

**Silvia Fernandez Villamil**

Argentina

**Ezio Ferroglio**

Italy

**Juliana Fietto**

Brazil

**Mark Fife**

United Kingdom

**Julio V Figueroa**

Mexico

**Deborah Finlaison**

Australia

**Katja Fischer**

Australia

**Peter Fischer**

United States

**Lucile Floeter-Winter**

Brazil

**Fernando Ivan Flores**

Mexico

**Éva Fok**

Hungary

**Gabor Foldvari**

Hungary

**Janet Foley**

United States

**Alessandra Fondati**

Italy

**Dina Fonseca**

United States

**Albin Fontaine**

France

**Mark Forbes**

Canada

**Christen Fornadel**

United States

**Jeremy Foster**

United States

**Pierre-Edouard Fournier**

France

**Antonio Frangipane Di Regalbono**

Italy

**Frits Franssen**

Netherlands

**Frederic Frezard**

Brazil

**Kevin Friesen**

Canada

**Ricardo Fujiwara**

Brazil

**Lisa Funkhouser-Jones**

United States

**Thomas Fürst**

United Kingdom

**Kanapathy Gajapathy**

Sri Lanka

**Kirill Galaktionov**

Russian Federation

**Eunice Galati**

Brazil

**Kurt Galbreath**

United States

**Montserrat Gállego**

Spain

**Claire Garros**

France

**Tini Garske**

United Kingdom

**Robin Gasser**

Australia

**Charles Gauci**

Australia

**R Gaugler**

United States

**Michael Gaunt**

United Kingdom

**Timothy Geary**

Canada

**Chris Geden**

United States

**Claudio Genchi**

Italy

**Solange Gennari**

Brazil

**David George**

United Kingdom

**Anuradha Ghosh**

United States

**Annunziata Giangaspero**

Italy

**Alessio Giannelli**

Italy

**Gabriella Gibson**

United Kingdom

**Wendy Gibson**

United Kingdom

**Nelly Gidaszewski**

France

**Julia Gillhuber**

Germany

**Kirsten Gillingwater**

Switzerland

**John Gimnig**

United States

**Howard Ginsberg**

United States

**Olivier Glaizot**

Switzerland

**Alyssa Gleichsner**

United States

**Geoffrey Gobert**

Australia

**Jerome Goddard**

United States

**Heidi Goethert**

United States

**Tony Goldberg**

United States

**Nicholas Golding**

United Kingdom

**Bruno Gomes**

Portugal

**Maria Angeles Gómez-Morales**

Italy

**Andres Gomez-Palacio**

Colombia

**Lilia Gonzalez-Ceron**

Mexico

**David Gorla**

Argentina

**Nicole Gottdenker**

United States

**Yuval Gottlieb**

Israel

**Bruno Gottstein**

Switzerland

**Louis Clement Gouagna**

Reunion

**Nicodem James Govella**

Tanzania

**Renaud Govoetchan**

Benin

**Daniel Grabner**

Germany

**Luigi Gradoni**

Italy

**Andrea Graham**

United States

**Robert Graham**

United Kingdom

**Jeremy Gray**

Ireland

**Marta Grech**

Argentina

**Christoph G Grevelding**

Germany

**Mario Grijalva**

United States

**Edmundo Grisard**

Brazil

**Libor Grubhoffer**

Czech Republic

**Liu Guangyuan**

China

**Pablo Guerenstein**

Argentina

**Jorge Guerrero**

United States

**Felipe Guhl**

Colombia

**Rodrigo Gurgel-Goncalves**

Brazil

**Alexander Gutfraind**

United States

**Paul Gwakisa**

Tanzania

**Milos Halán**

Slovakia

**Simon Hales**

New Zealand

**Carina Hall**

United States

**Sascha Hallett**

United States

**Katherine Halliday**

United Kingdom

**Omar Hamarsheh**

Palestinian Territory

**Gabriel Hamer**

United States

**Sarah Hamer**

United States

**Patrick Hamilton**

United Kingdom

**Edd Hammill**

Australia

**Qian Han**

United States

**Lee Hanlim**

Malaysia

**Ralph Harbach**

United Kingdom

**Laura Harburguer**

Argentina

**David Harley**

Australia

**William Harnett**

United Kingdom

**Caroline Harris**

United Kingdom

**Alan Harrison**

United Kingdom

**Leslie Harrison**

United Kingdom

**Sarah Hart**

United Kingdom

**Gunnar Hasle**

Norway

**Moawia Hassan**

Sudan

**John Hawdon**

United States

**Frances Hawkes**

United Kingdom

**Hadas Hawlena**

Israel

**Daniel Hegglin**

Switzerland

**Franz X Heinz**

Austria

**Emanuel Heitlinger**

Germany

**Joe Heitman**

United States

**Jesus Servando Hernandez-Orts**

Spain

**Luis Hernandez-Triana**

United Kingdom

**Claudia Patricia Herrera**

United States

**Geoff Hide**

United Kingdom

**Jeffrey Hii**

Australia

**Julian Hillyer**

United States

**Yousif Himeidan**

Sudan

**Barbara Hinney**

Austria

**Simone Hoeger**

Germany

**Ludwig Eduard Hoelzle**

Germany

**Anne Hoen**

United States

**Celia Holland**

Ireland

**Patricia Holman**

United States

**Peter Holmes**

United Kingdom

**Sung-Jong Hong**

Korea, South

**David Hoole**

United Kingdom

**Janet Hoole**

United Kingdom

**Petr Horak**

Czech Republic

**Sandor Hornok**

Hungary

**Linda Horspool**

Netherlands

**Olaf Horstick**

Germany

**John Horton**

United Kingdom

**Kees Emil Hovius**

Netherlands

**Gabriela Hrčkova**

Slovakia

**Min Hu**

China

**Shaomin Hu**

United States

**Xuemei Hu**

China

**Rodney Hull**

South Africa

**Hilary Hurd**

United Kingdom

**Eveline Hürlimann**

Switzerland

**Zuzana Hurnikova**

Slovakia

**Ana Hurtado**

Spain

**Ikuo Igarashi**

Japan

**Ralf Ignatius**

Germany

**Asma Iqbal**

Canada

**Seth Irish**

United States

**Peter Irwin**

Australia

**Jun Isoe**

United States

**Akira Ito**

Japan

**Arezki Izri**

France

**Abdul Jabbar**

Australia

**Isabel Jado**

Spain

**Thomas GT Jaenson**

Sweden

**Helen Victoria Jamet**

Tanzania

**Narissara Jariyapan**

Thailand

**Wojciech Jarosz**

Poland

**Musa Jawara**

Gambia

**Maamun Jeneby**

Kenya

**Per Moestrup Jensen**

Denmark

**Aaron Jex**

Australia

**Antonio Jiménez-Ruiz**

Spain

**Anja Joachim**

Austria

**Maria Vang Johansen**

Denmark

**Christopher Jones**

United Kingdom

**Frans Jongejan**

Netherlands

**Nick Jonsson**

United Kingdom

**Patricia Marta Juárez**

Argentina

**Claudia Julio**

Portugal

**Kerstin Junker**

South Africa

**Bilali Kabula**

Tanzania

**Szymon Kaczanowski**

Poland

**Lewis Kahn**

Australia

**Ramaswamy Kalyanasundaram**

United States

**Joshua Kamani**

Nigeria

**Joseph Kamau**

Kenya

**Colince Kamdem**

United States

**Helge Kampen**

Germany

**Ramaiah Kapa Dasaradha**

India

**Alex Karagiannis**

Switzerland

**Panagiotis Karanis**

Germany

**Afework Kassu**

Ethiopia

**Frank Katzer**

United Kingdom

**Shin-Ichiro Kawazu**

Japan

**Maria Kazimirova**

Slovakia

**Jennifer Keiser**

Switzerland

**Louise Kelly-Hope**

United Kingdom

**Malcolm Kennedy**

United Kingdom

**Noteila Khalid**

Sudan

**Solomon Kibret**

Ethiopia

**Linda Kidd**

United States

**Benson Kidenya**

Tanzania

**Gerry Killeen**

Tanzania

**A Marm Kilpatrick**

United States

**Jeong-Ho Kim**

Korea, South

**Carsten Kirkeby**

Denmark

**Shin-Ichi Kitamura**

Japan

**Jovin Kitau**

Tanzania

**Terry Klein**

United States

**Daniel Kline**

United States

**Stefanie Knopp**

Switzerland

**Dave Knox**

United Kingdom

**Tessa Knox**

Kenya

**Lizette Koekemoer**

South Africa

**Constantianus JM Koenraadt**

Netherlands

**Mathurin Koffi**

Cote D’ivoire

**Wan Koh**

Australia

**Andreas König**

Germany

**Petr Kopacek**

Czech Republic

**Danuta Kosik-Bogacka**

Poland

**Aneta Kostadinova**

Czech Republic

**Andrew Kotze**

Australia

**Benjamin Koudou**

United Kingdom

**Artemis Koukounari**

United Kingdom

**Laura Kramer**

Italy

**Boris Krasnov**

Israel

**Peter Krause**

United States

**Lena Krayter**

Germany

**Alison Krentel**

Canada

**Jürgen Krücken**

Germany

**Andreas Krueger**

Germany

**Olena Kudlai**

Lithuania

**Michael Kurth**

Germany

**Eliningaya Kweka**

Tanzania

**Marcelo Labruna**

Brazil

**Carlos Lanusse**

Argentina

**Nicole Laronde**

United States

**Brian Lassen**

Estonia

**Maria Stefania Latrofa**

Italy

**Yee-Ling Lau**

Malaysia

**Anne Laudisoit**

Belgium

**Claudio Lazzari**

France

**Stephen Leak**

United Kingdom

**Karin Lebl**

Austria

**Chow-Yang Lee**

Malaysia

**François Lefebvre**

France

**Tovi Lehmann**

United States

**David Leiby**

United States

**Paul Leisnham**

United States

**Alexandre Leitão**

Portugal

**David Leitsch**

Austria

**Meryem Lemrani**

Morocco

**Andres G Lescano**

Peru

**Edward Levri**

United States

**Michael Lewis**

United Kingdom

**Ala Lew-Tabor**

Australia

**Jianxu Li**

United States

**Jun Li**

United States

**Chenyi Li**

China

**Tiaoying Li**

China

**Xiangrui Li**

China

**You-Sheng Liang**

China

**Tamara Lima-Camara**

Brazil

**Lin Lin**

United States

**Paul Linser**

United States

**Yvonne Linton**

United States

**Susan Little**

United States

**Tim Littlewood**

United Kingdom

**Mingyuan Liu**

China

**Quan Liu**

China

**Lu Liu**

China

**Qun Liu**

China

**Jose Loaiza**

Panama

**Remo Lobetti**

South Africa

**Philippe Loiseau**

France

**James Lok**

United States

**Job Lopez**

United States

**Lena Lorenz**

United Kingdom

**Marcelo Lorenzo**

Brazil

**Vincenzo Lorusso**

United Kingdom

**Bertrand Losson**

Belgium

**Saul Lozano-Fuentes**

United States

**Shaohong Lu**

China

**Renke Lühken**

Germany

**Nongkran Lumjuan**

United Kingdom

**Zhao-Rong Lun**

China

**Jan Lundstrom**

Sweden

**Joel Lutomiah**

Kenya

**Zhiyue Lv**

China

**David Chien Lye**

Singapore

**Eugene Lyons**

United States

**Yajun Ma**

China

**Rafael Maciel-De-Freitas**

Brazil

**Charles Mackenzie**

United States

**Ewan Macleod**

United Kingdom

**Calum Macpherson**

Grenada

**Henry Madsen**

Denmark

**Yoshimasa Maeno**

Japan

**Carla Maia**

Portugal

**Flavia Maia Da Silva**

Brazil

**Silas Majambere**

Tanzania

**Peter Makaula**

Malawi

**Benjamin Makepeace**

United Kingdom

**Joanna Makol**

Poland

**Imna Malele**

Tanzania

**Elfatih Malik**

Sudan

**Francesca Mancianti**

Italy

**Sven Mangelinckx**

Belgium

**Sylvie Manguin**

France

**Alphaxard Manjurano**

Tanzania

**Rajinder Mann**

United States

**Madhumita Manna**

India

**Arlei Marcili**

Brazil

**Mara Mariconti**

Italy

**Osvaldo Marinotti**

United States

**Coralie Martin**

France

**Josué Martínez-De La Puente**

Spain

**Ademir J Martins**

Brazil

**Gustavo Martins**

Brazil

**Virginia Marugan-Hernandez**

United Kingdom

**Santiago Mas-Coma**

Spain

**Peter Mason**

Zimbabwe

**Alessandro Massolo Massolo**

Canada

**Susan Masta**

United States

**Justin Masumu**

Congo, Democratic Republic

**Alexander Mathis**

Switzerland

**Olga Matos**

Portugal

**Johnson Matowo**

Tanzania

**Kotaro Matsumoto**

Japan

**Sonja Matthee**

South Africa

**Aaron Maule**

United Kingdom

**Patrick Mavingui**

France

**Camila Mazzoni**

Germany

**Leonard Mboera**

Tanzania

**Charles Mbogo**

Kenya

**Jere Mcbride**

United States

**Karen D Mccoy**

France

**Kathryn Mcgovern**

United States

**Elizabeth Mcgraw**

Australia

**Bradford Mcgwire**

United States

**Samantha Mcnulty**

United States

**Andy Mead**

United States

**Alexandra Ivo Medeiros**

Brazil

**Jolyon Medlock**

United Kingdom

**Sergei Medvedev**

Russian Federation

**Janet Meredith**

United Kingdom

**Catherine Merrick**

United Kingdom

**Louisa Messenger**

United Kingdom

**Gretchen Messick**

United States

**Janet Midega**

Kenya

**Ewa Mierzejewska**

Poland

**Andrei Daniel Mihalca**

Romania

**Thomas Miller**

United States

**Caroline Millins**

United Kingdom

**Noboru Minakawa**

Japan

**Dennis Minchella**

United States

**Tiago Mineo**

Brazil

**Haris Mirza**

United States

**Nicolas Moiroux**

France

**Norhayati Moktar**

Malaysia

**Goudarz Molaei**

United States

**Ricardo Molina**

Spain

**David Molyneux**

United Kingdom

**Federica Monaco**

Italy

**Dinesh Mondal**

Bangladesh

**Fernando Monteiro**

Brazil

**Karina Monteiro**

Brazil

**Victor Monteon**

Mexico

**Ronald Morales Vargas**

Thailand

**Jorge Morales-Montor**

Mexico

**Christopher Mores**

United States

**Neil Morley**

United Kingdom

**Liam Morrison**

United Kingdom

**Andy Morse**

United Kingdom

**Karine Mouline**

France

**Catherine Moyes**

United Kingdom

**Dennis Muhanguzi**

Uganda

**Paolo Mulatti**

Italy

**Albert Mulenga**

United States

**Pie Müller**

Switzerland

**Ulrike G Munderloh**

United States

**Stephen Munga**

Kenya

**Givemore Munhenga**

South Africa

**Miguel A Muniain**

Spain

**Maria Anice Mureb Sallum**

Brazil

**Carlos Muskus**

Colombia

**Lizzy Mwamburi**

Kenya

**Beda John Mwang’onde**

Tanzania

**Pauline Mwinzi**

Kenya

**Jeannie N Dos Santos**

Brazil

**Byoung-Kuk Na**

Korea, South

**Steven Nadler**

United States

**Minoru Nakao**

Japan

**Ryo Nakao**

Japan

**Gikonyo Nicholas**

Kenya

**Pin Nie**

China

**Martin Nielsen**

United States

**Birgit Nikolay**

United Kingdom

**Kenneth Nilsson**

Sweden

**Alasdair Nisbet**

United Kingdom

**Sammy Njenga**

Kenya

**Flobert Njiokou**

Cameroon

**Doris Njomo**

Kenya

**Steven Norris**

United States

**Francesca Notarangelo**

United States

**Eva Nováková**

Czech Republic

**Adam Novobilský**

Sweden

**Patricia Nuttall**

United Kingdom

**Philippe Nwane**

Cameroon

**Marcos Obara**

Brazil

**Eric Ochomo**

Kenya

**Peter Odermatt**

Switzerland

**Nick Ogden**

Canada

**Oivind Oines**

Norway

**Fredros Okumu**

Tanzania

**Evelyn Olanga**

Kenya

**Pedro Oliveira**

Brazil

**Samantha O’loughlin**

United Kingdom

**Annette Olsen**

Denmark

**Peter Olson**

United Kingdom

**Laura Ordeix**

Spain

**M Guadalupe Ortega-Pierres**

Mexico

**Jose A Oteo**

Spain

**Joseph O’tousa**

United States

**Domenico Otranto**

Italy

**Jean Pierre Nableni Ouattara**

Belgium

**Richard Martin Oxborough**

United Kingdom

**Krijn Paaijmans**

United States

**Kevin Palmer**

United States

**Weiqing Pan**

China

**Rosario Panadero**

Spain

**Nenad Pandak**

Croatia

**Ingrid Papajova**

Slovakia

**Roberto Papini**

Italy

**Claudia Paredes**

Spain

**Philippe Parola**

France

**Parviz Parvizi**

Iran

**Luiz Felipe Passero**

Brazil

**Julien Pelletier**

United Kingdom

**Pamela Pennington**

Guatemala

**Maria Grazia Pennisi**

Italy

**Adalberto Pérez De León**

United States

**Mercedes Pérez-Ruiz**

Spain

**Ricardo Perez-Sanchez**

Spain

**Alex Perkins**

United States

**Eskild Petersen**

Denmark

**Dusan Petric**

Serbia

**Kurt Pfister**

Germany

**David Pigott**

United Kingdom

**Matthew Playford**

Australia

**Philippe Poirier**

France

**Marco Pombi**

Italy

**Loganathan Ponnusamy**

United States

**Aránzazu Portillo**

Spain

**Robert Poulin**

New Zealand

**Christine Pourcel**

France

**Bobbi Pritt**

United States

**Anna Protasio**

United Kingdom

**Anna Psaroulaki**

Greece

**Concepcion Puerta**

Colombia

**Odile Puijalon**

France

**Rachel Pullan**

United Kingdom

**Moneeb Qablan**

Czech Republic

**Men-Bao Qian**

China

**Daofeng Qu**

China

**Martha Lucia Quiñones**

Colombia

**Sonia Mara Raboni**

Brazil

**Fara Nantenaina Raharimalala**

Belgium

**Marcelo Ramalho-Ortigao**

United States

**Urvashi Ramphul**

United States

**Reda Ramzy**

Egypt

**Sarah Randolph**

United Kingdom

**Hilary Ranson**

United Kingdom

**Didier Raoult**

France

**Vincent Raquin**

France

**Jason Rasgon**

United States

**Amy Rasley**

United States

**Norman Ratcliffe**

United Kingdom

**Jean - Baptiste Rayaisse**

Burkina Faso

**Paul Ready**

United Kingdom

**Eduardo Rebollar-Tellez**

Mexico

**Steffen Rehbein**

Germany

**Craig Reinemeyer**

United States

**William Reisen**

United States

**Michael Reiskind**

United States

**Dinglasan Rhoel**

United States

**Paulo Ribolla**

Brazil

**Allen Richards**

United States

**Stephanie Richards**

United States

**Michael Riehle**

United States

**Laura Rinaldi**

Italy

**Jacob Riveron**

United Kingdom

**Myriam Riyad**

Morocco

**Annapaola Rizzoli**

Italy

**Bruno Roatt**

Brazil

**Wojciech Rode**

Poland

**Boris Rodenko**

United Kingdom

**David Roiz**

Spain

**Antonieta Rojas-De-Arias**

Paraguay

**David Rollinson**

United Kingdom

**Christine Romana**

France

**Gustavo Romero**

Brazil

**Benjamin Rosenthal**

United States

**Luca Rossi**

Italy

**Xavier Roura**

Spain

**Mark Rowland**

United Kingdom

**Nataliia Rudenko**

Czech Republic

**James Rudge**

Thailand

**Karla Saavedra-Rodriguez**

United States

**Martha Saboya**

United States

**Farhad Safarpoor Dehkordi**

Iran

**Angel Sainz**

Spain

**Dan Salkeld**

United States

**Oscar Salomon**

Argentina

**Richard Samuels**

Brazil

**Ana Sanchez**

Canada

**Christopher Sanders**

United Kingdom

**Fiona Sansom**

Australia

**Diego Santiago-Alarcon**

Mexico

**Huarrisson Santos**

Brazil

**Mirella Santos**

Brazil

**Pierre Sasal**

French Polynesia

**Yukita Sato**

Japan

**Fadjar Satrija**

Isle Of Man

**Hirofumi Sawa**

Japan

**Patricia Scaraffia**

United States

**Vera Margarete Scarpassa**

Brazil

**Bettina Schelkle**

United Kingdom

**Justin Schmidt**

United States

**Manuela Schnyder**

Switzerland

**Gabriele Schoenian**

Germany

**Joerg D Schulzke**

Germany

**Megan Schwartz**

United States

**Diana Scorpio**

Saint Kitts And Nevis

**W Evan Secor**

United States

**Luigi Sedda**

United Kingdom

**Jennifer Seddon**

Australia

**Richard Selby**

United Kingdom

**Juan Carlos Sepulveda-Arias**

Colombia

**Renfu Shao**

Australia

**Igor Sharakhov**

United States

**Jeffrey Shaw**

Brazil

**Jilong Shen**

China

**Yang Shen**

United States

**Jeffrey Sherman**

United States

**Clive Shiff**

United States

**Paloma Shimabukuro**

Brazil

**Techalew Shimelis**

Ethiopia

**Sung-Shik Shin**

Korea South

**Barbara Shock**

United States

**Mark Edward Siddall**

United States

**Cornelia Silaghi**

Switzerland

**Katja Silbermayr**

Austria

**Maria Helena Silva-Filha**

Brazil

**Frederic Simard**

Burkina Faso

**Heven Sime**

Ethiopia

**Andrea Simkova**

Czech Republic

**Gustave Simo**

Cameroon

**Steven Singer**

United States

**Neeloo Singh**

India

**Shanker Singh**

India

**Sarman Singh**

India

**Upinder Singh**

United States

**Pavel Siroky**

Czech Republic

**Joyce Siwila-Saasa**

Zambia

**Rebecca Skalsky**

United States

**Barton Slatko**

United States

**Michel Slotman**

United States

**Nicholas Smith**

Australia

**Todd Smith**

Canada

**Daniel Snyder**

United States

**Rodrigo Soares**

Brazil

**Ljiljana Sofronic-Milosavljevic**

Serbia

**Philippe Solano**

Burkina Faso

**Laia Solano-Gallego**

Spain

**Krzysztof Solarz**

Poland

**Pradya Somboon**

Thailand

**Kerry Sondgeroth**

United States

**Marcos Sorgine**

Brazil

**Ketty Soteriadou**

Greece

**Manuel Soto**

Spain

**Jose Carlos Sousa Figueiredo**

Angola

**Maria A Souza**

Brazil

**Sabine Specht**

Germany

**Eva Spitalska**

Slovakia

**Hein Sprong**

Netherlands

**Banchob Sripa**

Thailand

**Joanna Stanczak**

Poland

**Claire Standley**

United States

**Michal Stanko**

Slovakia

**Damien Stark**

Australia

**Michael Stear**

United Kingdom

**Peter Steinmann**

Switzerland

**Christen Rune Stensvold**

Denmark

**Dietmar Steverding**

United Kingdom

**Eileen Stillwaggon**

United States

**Ann-Kathrin Stock**

Germany

**Krzysztof Stojecki**

Poland

**Wilma Stolk**

Netherlands

**Russell Stothard**

United Kingdom

**Reinhard Straubinger**

Germany

**Clare Strode**

United Kingdom

**Ellen Stromdahl**

United States

**Snorre Stuen**

Norway

**Chunlei Su**

United States

**Xin-Zhuan Su**

United States

**Gopinath Subash**

Malaysia

**Hiromu Sugiyama**

Japan

**Michael Sukhdeo**

United States

**Bindu Sukumaran**

Singapore

**Xun Suo**

China

**Bernd Sures**

Germany

**Wannapa Suwonkerd**

Thailand

**Gerardo Suzán**

Mexico

**Din Syafruddin**

Indonesia

**Matias Szabo**

Brazil

**Csaba Székely**

Hungary

**Yasuhiro Takashima**

Japan

**Petrus Tang**

Taiwan

**D’almeida Tania Carenne D A**

France

**Dingyin Tao**

United States

**Xinrong Tao**

United States

**Sun Tee Tay**

Malaysia

**Mark Taylor**

United Kingdom

**David Poumo Tchouassi**

Kenya

**Marta Teixeira**

Brazil

**Sam Telford**

United States

**Erich Telleria**

Brazil

**Lesly Temesvari**

United States

**Olle Terenius**

Sweden

**Carlos Termignoni**

Brazil

**Saravanan Thangamani**

United States

**Andrew Thompson**

Australia

**Li-Guang Tian**

China

**Michel Tibayrenc**

Thailand

**Ellen Tijsse-Klasen**

Netherlands

**Swati Tiwari**

India

**Dan Tompkins**

New Zealand

**Miray Tonk**

Czech Republic

**Steve Torr**

United Kingdom

**Harold Townson**

United Kingdom

**Donato Traversa**

Italy

**Omar Triana**

Colombia

**Michele Trotta**

Italy

**Jean Tsao**

United States

**Takashi Tsunoda**

Japan

**Patrick Tungu**

Tanzania

**Salvatore Turco**

United States

**Julio Turrens**

United States

**Rahul Tyagi**

United States

**Kevin Tyler**

United Kingdom

**Florencio Ubeira**

Spain

**Joseph Urban**

United States

**Igor Uspensky**

Israel

**Juerg Utzinger**

Switzerland

**ETellervo Valtonen**

Finland

**Wim van Bortel**

Sweden

**Lisette van Lieshout**

Netherlands

**Giancarlo Vanini**

United States

**Antonio Varcasia**

Italy

**Jefferson Vaughan**

United States

**Muriel Vayssier-Taussat**

France

**Ana Vázquez González**

Spain

**Thirumalaisamy Velavan**

Germany

**Luigi Venco**

Italy

**V Venkatesalu**

India

**Gert Venter**

South Africa

**Sergio Verjovski-Almeida**

Brazil

**Guilherme Verocai**

Canada

**Jaco Verweij**

Netherlands

**Elvina Viennet**

Australia

**Sharon Yvette Angelina Villanueva**

Philippines

**Delespaux Vincent**

Belgium

**Joseph Vinetz**

United States

**Parnpen Viriyavejakul**

Thailand

**Stalin Viswanathan**

India

**Christopher Vitek**

United States

**Lea Vojta**

Croatia

**Petr Volf**

Czech Republic

**John Vontas**

Greece

**Jan Votypka**

Czech Republic

**Indra Vythilingam**

Malaysia

**Sabina Wachira**

Kenya

**Andrea Waeschenbach**

United Kingdom

**David M Wagner**

United States

**Jitra Waikagul**

Thailand

**Jessica Waite**

United States

**Cheryl Waldner**

Canada

**Martin Walker**

United Kingdom

**Anthony Walker**

United Kingdom

**Edward Walker**

United States

**Richard Wall**

United Kingdom

**Julia Walochnik**

Austria

**Shelley Walton**

Australia

**Shanqing Wang**

China

**Zhong-Quan Wang**

China

**Xiaoyun Wang**

United States

**Samuel Wanji**

Cameroon

**Michael Ward**

United States

**Dorn Watthanakulpanich**

Thailand

**Haylee Weaver**

Australia

**Cameron Webb**

Australia

**Bonnie Webster**

United Kingdom

**David Weetman**

United Kingdom

**Susan Welburn**

United Kingdom

**Renata Welc-Faleciak**

Poland

**Gareth Westrop**

United Kingdom

**Stephen Wikel**

United States

**Craig Wilding**

United Kingdom

**Richard Wilkerson**

United States

**Craig Williams**

Australia

**David Williams**

United States

**Roderick Williams**

United Kingdom

**Trevor Williams**

Mexico

**Mark Wilson**

United Kingdom

**Michael David Wilson**

Ghana

**R Alan Wilson**

United Kingdom

**Denis Wolf**

Germany

**Charles Wondji**

United Kingdom

**Susan J Wong**

United States

**Jacklyn Wong**

United States

**Hai-Wei Wu**

United States

**Zhiyong Xi**

United States

**Lihua Xiao**

United States

**Maria De Fátima Ximenes**

Brazil

**Rui-De Xue**

United States

**Laith Yakob**

United Kingdom

**Tsuyoshi Yamaguchi**

Japan

**Teresa K Yamana**

United States

**Guiyun Yan**

United States

**Tetsuya Yanagida**

Japan

**Kun Yang**

China

**Tingbao Yang**

China

**Jifei Yang**

China

**Peiling Yap**

Switzerland

**Chuan-Min Yen**

Taiwan

**Kor Yereli**

Turkey

**Delenasaw Yewhalaw**

Ethiopia

**Doudou Yobi**

Congo, Democratic Republic

**Nobuko Yoshida**

Brazil

**Simon Young**

United Kingdom

**Xinbing Yu**

China

**Vyacheslav Yurchenko**

Czech Republic

**Arnaldo Zaha**

Brazil

**Stefania Zanet**

Italy

**Alia Zayed**

Egypt

**Pavel Zehtindjiev**

Bulgaria

**Bin Zhan**

United States

**Tongyan Zhao**

China

**Guanghui Zhao**

China

**Junlong Zhao**

China

**Elyes Zhioua**

Tunisia

**Kun Yan Zhu**

United States

**Xing-Quan Zhu**

China

**Jinsong Zhu**

United States

**Xinping Zhu**

China

**Thomas Zoller**

Germany

**Ines Zulantay**

Chile

**Ludek Zurek**

United States

**Erich Zweygarth**

Germany

